# Effect of vitamin D supplementation on brain waves, behavioral performance, nitric oxide, malondialdehyde, and high-sensitivity C-reactive protein in children with attention deficit/hyperactivity disorder: study protocol for a randomized clinical trial

**DOI:** 10.1186/s13063-022-06837-1

**Published:** 2022-10-22

**Authors:** Abbas Ali Sangouni, Hamid Mirhosseini, Mahdieh Hosseinzadeh

**Affiliations:** 1 Nutrition and Food Security Research Center, Shahid Sadoughi University of Medical Sciences, Yazd, Iran; 2 Department of Nutrition, School of Public Health, Shahid Sadoughi University of Medical Sciences, Yazd, Iran; 3 Research Center of Addiction and Behavioral Sciences, Shahid Sadoughi University of Medical sciences, Yazd, Iran; 4 Student Research Committee, Shahid Sadoughi University of Medical Sciences, Yazd, Iran

**Keywords:** Attention deficit/hyperactivity disorder, Vitamin D, Brain waves, Behavioral performance, Oxidative stress

## Abstract

**Background:**

Attention deficit/hyperactivity disorder (ADHD) is the most common chronic mental and behavioral disorder among children. Some studies showed the lower levels of vitamin D in patients with ADHD compared with the healthy people. Few clinical trials were conducted in this field. The present study will be performed to examine the effect of vitamin D supplementation in children with ADHD.

**Methods:**

We will conduct a double-blind, randomized controlled clinical trial to investigate the effect of vitamin D supplementation on brain waves, behavioral performance, serum nitric oxide, malondialdehyde, and high-sensitivity C-reactive protein in 50 patients with ADHD. The intervention group will receive one capsule 50,000 IU vitamin D every week, for 8 weeks. The control group will receive one placebo capsule containing 1000 mg olive oil every week. Electroencephalography will be performed for 10 min using Brain Master Discovery from 19 scalp sites both before the first intervention and the 10 sessions of the therapy. The artifact-free periods of 1-min electroencephalography data will be analyzed for quantitative electroencephalography measures.

**Discussion:**

For the first time, this clinical trial will evaluate the effect of vitamin D supplementation on brain waves, serum nitric oxide, malondialdehyde, and high-sensitivity C-reactive protein in patients with ADHD. The results of the present clinical trial will provide a better vision about the vitamin D efficacy in patients with ADHD.

**Trial registration:**

Registered on 5 November 2020 at Iranian Registry of Clinical Trials with code number IRCT20200922048802N1 (https://www.irct.ir/trial/51410).

## Background

Attention deficit/hyperactivity disorder (ADHD) is one of the most common neurocognitive behavioral disorders in children [1]. The main symptoms of ADHD are hyperactivity, impulsivity, and inattention, and it is also associated with psychiatric disorders such as autism, anxiety, and oppositional defiant disorder (ODD) [2, 3]. The global prevalence of ADHD is 6–7% [4]. Neurotransmitters dopamine and serotonin have a critical role in attention, concentration, and other cognitive functions [5]. Evidence revealed disorders in the dopaminergic system of basal ganglia in ADHD children [5, 6]. In addition, ADHD is accompanied with spectral changes in subject brain waves including the higher rate of delta and theta values power spectra as well as the theta/beta and theta/alpha ratios of the frontal and central areas and the reduced power of sensorimotor rhythm (SMR) in the central and motor cortices [7, 8]. Although the pathophysiology is not fully clear, oxidative stress and inflammation are linked to ADHD [9, 10]. Levels of malondialdehyde (MDA), which is a marker of oxidative stress, is high among patients with ADHD [11]. In addition, patients with ADHD have lower levels of antioxidant enzymes [12, 13]. It has been suggested that nitric oxide (NO), which is an important antioxidant molecule for the proper function of the central nervous system, has a functional role in learning and memory [14–16]. On the other hand, cytokines have a critical role in the metabolism of tryptophan and dopaminergic pathways in the brain [10, 17]. Patients with ADHD have higher levels of pro-inflammatory markers and lower levels of the anti-inflammatory cytokine interleukin 4 (IL-4) in the cerebrospinal fluid [18]. Likewise, higher serum level of inflammatory markers is associated with higher ADHD symptom severity [19]. It has been reported that elevated levels of C-reactive protein (CRP), which is a main marker of inflammation in clinical practice, is associated with impairments in memory, learning ability, and mental flexibility [20, 21].

Central nervous system (CNS) stimulants like methylphenidate and amphetamine are prescribed for patients with ADHD [22]. However, CNS stimulants have various side effects such as arrhythmia, insomnia, irritability, and decreased appetite, and the rate of compliance to this treatment is not high [23–25]. In addition, atomoxetine and alpha-2-agonists (like clonidine and guanfacine), which are non-stimulant drugs for ADHD, have several side effects including decreased appetite, vomiting, nausea, diarrhea, dry mouth, somnolence, fatigue, mood swings, dizziness, and constipation [23, 26]. Moreover, 30% of children with ADHD do not respond to drug treatments [22, 27]. The recent evidence suggested the beneficial effects of vitamin D on neurocognitive behavioral disorders [28, 29]. Vitamin D is a fat-soluble vitamin as well as a steroid hormone and acts in various organs of the body by binding to the vitamin D receptor (VDR) [30–32]. High levels of the VDR as well as 1*α*-hydroxylase enzyme (activator of vitamin D) in the brain suggest that vitamin D probably has physiological functions in the brain [33]. Serotonin, which is synthesized in the brain, is regulated by vitamin D [34]. Furthermore, vitamin D increases the expression of tyrosine kinase as an enzyme involved in the dopamine synthesis [35]. In addition, vitamin D has fewer side effects compared to other treatments for ADHD [23, 36]. Therefore, the compliance rate to treatment with vitamin D can be higher than treatment with common drugs for ADHD. Vitamin D can improve oxidative stress and inflammation via reducing the production of inflammatory markers such as interleukin-6 (IL-6), and tumor necrosis factor-α (TNF-α), inhibiting the proliferation of pro-inflammatory cells, increasing the expression of antioxidants and anti-inflammatory cytokines, attenuating endoplasmic reticulum stress, and decreasing mitochondrial dysfunctions [37–40].

A study found that higher level of vitamin D in pregnant women reduces risk of ADHD-like symptoms in the offspring [41]. Furthermore, some studies showed the lower levels of vitamin D in patients with ADHD compared with the healthy controls [6, 42, 43]. A meta-analysis of observational studies confirmed lower levels of vitamin D in patients with ADHD [44]. Two clinical trials evaluated the effect of vitamin D supplementation on the behavioral performance of children with ADHD and showed inconsistent results [45, 46]. Moreover, there is no clinical trial evaluating the effect of vitamin D on brain waves, oxidative stress and inflammation in patients with ADHD. The present study was designed to examine the effect of vitamin D supplementation on brain waves, behavioral performance, serum NO, MDA, and high sensitivity CRP (hs-CRP) in children with ADHD.

## Methods

### Study design

**Fig. 1:**
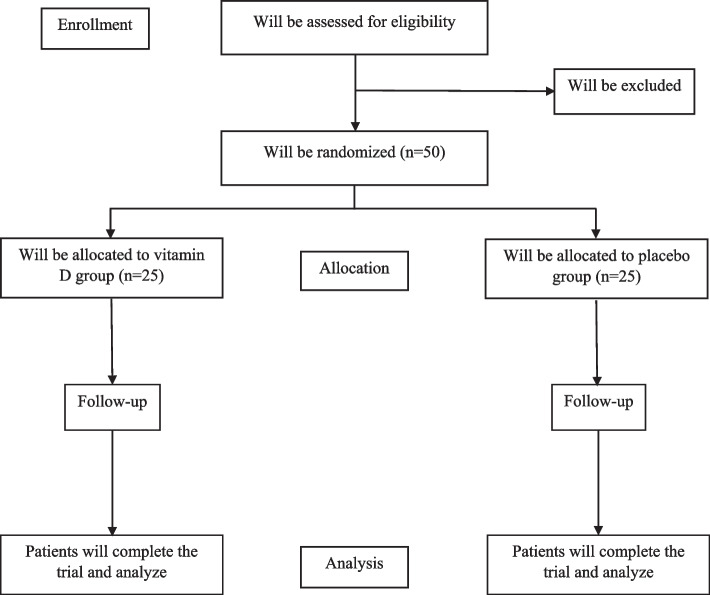
Flow chart of the study protocol

This study will be performed as a randomized double-blind placebo-controlled clinical trial (RCT) to investigate the effect of vitamin D supplementation for 8 weeks on brain waves, behavioral performance, NO, MDA, and hs-CRP in children with ADHD. The primary outcomes of the present study are the status of brain waves and the severity of ADHD symptoms. We will also evaluate serum NO, MDA, and hs-CRP as secondary outcomes. The present study protocol is derived from two studies that designed as the student theses with titles: [1] the effect of vitamin D supplementation on the brain mapping and behavioral performance of children with ADHD: a double-blind randomized controlled trial and [2] the effect of vitamin D supplementation on serum levels of MDA, NO, and hs-CRP in children with ADHD. The flow chart of study protocol is presented in Fig. 1.

### Objectives

Primary objectives are as follows:

- Compare the status of brain waves between the 2 groups and within each group, before and after intervention.
- Compare the mean ADHD symptoms severity between the 2 groups and within each group, before and after intervention.

In addition, the following objectives are the secondary objectives of the study:

- Compare the mean serum hs-CRP between the 2 groups and within each group, before and after intervention.
- Compare the mean of serum MDA, between the 2 groups and within each group, before and after intervention.
- Compare the mean of serum NO, between the 2 groups and within each group, before and after intervention.

### Participants

Participants will be 50 patients with ADHD. Patients will be recruited from Imam-Hossein psychological services center affiliated with Shahid Sadoughi University of Medical Sciences, Yazd, Iran. The inclusion criteria will be as follows: age 7–13 years, ADHD diagnosed by a psychiatrist, IQ higher than 80, theta brain waves higher than 7 Hz, beta brain waves lower than 13 Hz, increasing theta/beta power ratio in frontal, temporal and central electrode sites, resident of Yazd city, and informed consent signed by one of the patient’s parents. Exclusion criteria will be as follows: other psychotic diseases, taking any medication for ADHD during the past year, hormonal disorders including Addison's disease, Cushing syndrome, hyperparathyroidism, hypo- and hyperthyroidism, chronic diseases such as cancer, diabetes, stroke, kidney disease, liver disease, multiple sclerosis, fibromyalgia, and Parkinson’s disease, addiction of parents to alcohol/drugs at the time of the child’s birth, adherence to any specific diet in the past year, taking multivitamins or mineral supplements more than twice a month in the last 6 months, being on a special diet during the past year, and unwillingness to continue the study. We measured serum concentration of vitamin D before study. This study will be conducted among patients with ADHD who have vitamin D deficiency and vitamin D insufficiency.

### Ethics and trial registration

Participants and their parents will be notified about the risks and advantages of the present study, and then the investigator will ask them to sign an informed written consent. The study protocol and informed written consent are approved by the ethical committee of Shahid Sadoughi University of Medical Sciences and Health Services in Yazd, Iran (there are two theses with code number: IR.SSU.SPH.REC.1400.028 and also code number: IR.SSU.SPH.REC.1395.97). Any changes in the protocol that may affect the implementation of the study, including changes in study objectives, study design, participant population, sample size, and study procedures must be approved by the ethical committee of Shahid Sadoughi University of Medical Sciences and Health Services to be applicable. Before conducting a possible ancillary study, the approval of the ethics committee as well as the informed written consent of all participants will be obtained. Additional serum samples will be stored for use in future studies. The Shahid Sadoughi University of Medical Sciences will pay for the treatment of immediate adverse events related to study procedures. The registration of the study protocol was performed at the Iranian Registry of Clinical Trials with code number website, under code number IRCT20200922048802N1 with URL: https://www.irct.ir/trial/51410.

### ADHD diagnosis

According to the Diagnostic and Text Revision of Statistical Manual of Mental Disorders-Fifth Edition (DSM-V) [47, 48], the diagnosis of ADHD will be done by a psychiatrist. Based on the DSM-V criteria, a person who has at least six of the listed symptoms of inattention or symptoms of hyperactivity–impulsivity persisting for at least 6 months which are disruptive and inappropriate for the person’s developmental level is defined as a patient with ADHD [47, 48].

### Sample size

Sample size is calculated based on means and standard deviations for Conners’ ADHD Index, as the primary outcome of the study of Gow et al. [49] by considering 95% confidence interval, 80% power (*α* = 0.05 and *β* = 0.2), and using the following formula:

$$n=\frac{{\left(\textrm{z}\frac{\alpha }{2}+\textrm{z}\upbeta \right)}^2\times 2{S}^2\ }{{\left(x1-x2\right)}^2}$$

Considering a drop-out rate of approximately 10%, the final sample size needed was estimated to be 25 per group. All participants will be included in the RCT if they meet the inclusion criteria and are willing to participate in the study, until the estimated sample size is completed.

### Randomization and blinding

Simple (unrestricted) randomization will be performed, and participants will be divided randomly into two groups including intervention (vitamin D, *n* = 25) and control (placebo, *n* = 25) by a person who is not involved in the trial using the computer-generated random number table (produced by random allocation software) [50]. Concealing allocation sequence from those assigning participants to the intervention groups will be performed utilizing opaque sealed envelopes. Boxes of vitamin D or placebo will be named as A and B by a third person that is not involved in the study. Participants, investigators, and laboratory staff will be blinded to the treatment allocation. Randomization codes will be unlocked only after all patients complete the study protocol.

### Intervention

The intervention group will receive one capsule 50,000 IU vitamin D every week, for 8 weeks. The control group will receive one placebo capsule containing 1000 mg olive oil every week. Preparing placebo in the same appearance, taste, and color as the vitamin D will be performed by the Pharmacy Faculty of Shahid Sadoughi University of Medical Sciences. Participants will not receive any medication for ADHD during follow-up. Vitamin D and placebo will be packed in boxes with the same color, shape, and size. A person who is unaware about the trial details will label the boxes containing vitamin D and placebo as A or B. To preserve the blindness in case of serious adverse events, the third party will use unique codes. Every 4 weeks, the participants will receive the boxes of capsules. Each box contains 4 capsules (providing one capsule per week for 4 weeks). Participants must consume the contents of each box within 4 weeks. At the end of the study, the person who will label the boxes will inform the researchers about details of labeling.

### Adherence

Vitamin D and placebo boxes will be given to participants at weeks 0 and 4. Participants will be asked to return the boxes with their remaining contents at the end of every 4 weeks, and any occurrence of adverse events will be recorded. To evaluate the compliance rate, the consumption of vitamin D and placebo will be monitored at the end of each 4 weeks of intervention. At the end of intervention, the remaining contents of bottles will be recorded for each participant. Consuming less than 80% of the administered vitamin D or placebo will be defined as poor compliance. Participants with poor compliance will be excluded from study and their data will not be analyzed at the end of the study. After the study, the participant’s results will be provided to the participant's parents. Before the final data collection, parents will be contacted by phone to remind them. Participants can leave the study for any reason at any time. The investigator can withdraw participants from the trial (after consultation with the protocol chair and ethical committee) in order to protect the safety of participants or if they are unwilling or unable to continue the study. All randomized participants who are discontinued from study will follow the same schedule of events as those participants who continue the study except adherence assessment.

### Data collection

#### ADHD symptoms assessment

In the assessment of ADHD symptoms, Conners’ behavior rating scale which is a clinical tool evaluating child behavioral disorders will be used. Conners’ rating scale is divided into three categories, which are determined by the person who filled it (parent, teacher and self-report). For this trial, Conners’ parent rating scale (CPRS) [51] will be used. CPRS checklist makes parental reports of the children’s basic problems in the setting of outpatient psychiatry. The CPRS includes questions about different aspect of patients’ problems and shows different type of ADHD symptoms [51]. Conners’ questions gives ADHD Conners scores that indicates the severity of ADHD symptoms. In addition, all questions can be divided into three subscales by symptoms including inattention, hyperactivity/impulsivity, and combination type.

#### Electroencephalography

Electroencephalography (EEG) will be performed for 10 min using Brain Master Discovery from 19 scalp sites both before the first intervention and the 10 sessions of the therapy. The artifact-free periods of 1-min EEG data will be analyzed for quantitative EEG (QEEG) measures using the Neuroguide software. Background rhythm in relative power of theta (4–7.5 Hz), alpha (8–12 Hz) of the frontal and central electrodes, and the theta/beta and theta/alpha ratios in motor cortices will be analyzed by the SPSS software version 24.

#### Dietary intake and physical activity assessment

To evaluate dietary intake of subjects, a 3-day (1 weekend day and 2 nonconsecutive weekdays) food recall will be used at weeks 0, 4, and 8. The short form of International Physical Activity Questionnaire (IPAQ) [52] will be used for assessment of physical activity at weeks 0, 4, and 8.

#### Anthropometric evaluations

Height will be measured in standing position at weeks 0 and 8, using a non-stretched tape with an accuracy of 0.5 cm. Weight will be measured based on standard protocols with light clothes and without shoes by seca scale with an accuracy of 100 g. Body mass index (BMI) will be calculated by the following formula: weight (kg)/height squared (m^2^).

#### Laboratory assessments

Laboratory assessments will be performed at weeks 0, and 8. 5 cc blood will be drawn after 12 h fasting and will be centrifuged for 10 min at a speed of 3600 rpm. Serum samples poured into the microtubes will be immediately frozen at − 80 ° C. NO, MDA, and hs-CRP will be measured by ELISA method using Q-1-DiaPlus, USA, kits. In addition, using chemiluminescent microparticle immunoassay (CMIA) method, measuring the serum concentration of vitamin D will be performed. Serum calcium and phosphate will be measured by Biosystems, Barcelona, Spain, kits using colorimetric analysis method. Measurements will be performed based on standard methods.

#### Data management

Data will be entered electronically at the participating site. In addition, the forms will be kept on file at the participating site. Personnel at the participating site will send the copy of forms (files) to data coordinating center (DCC). The DCC will send weekly email reports about missing data, missing forms, and missing visits. Personnel at the participating site will review these reports for accuracy. The DCC will monitor source documents and will conduct at least one on-site monitoring visit during the study. DCC's primary objectives during site visits are to educate, support, and resolve problems. This process will be independent from investigators. Participant files will be maintained for a period of 2 years after completion of the trial.

#### Safety and adverse effects

**Fig. 2:**
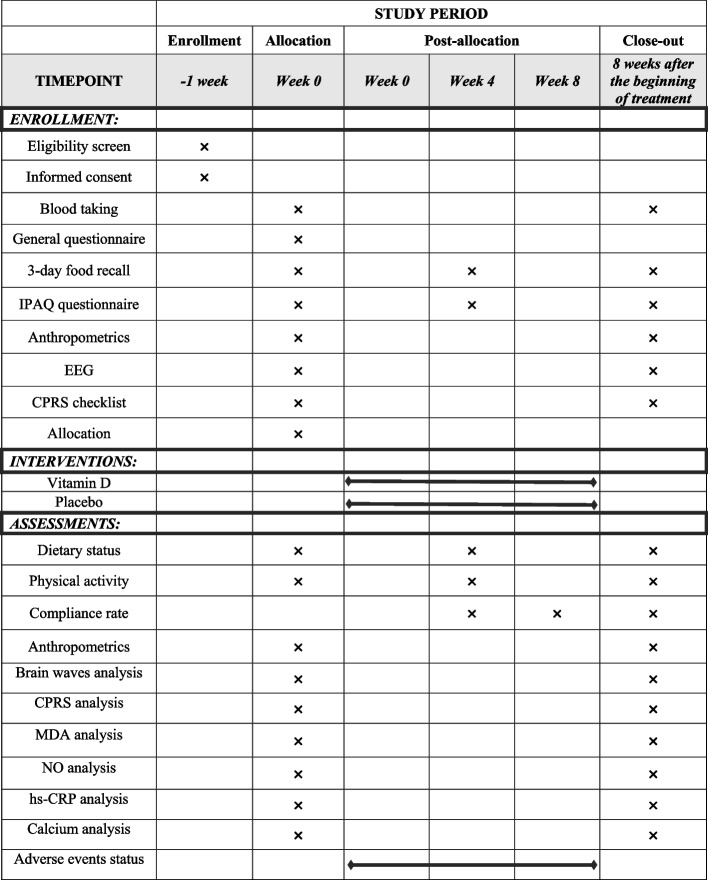
Schedule for enrollment, intervention, and assessment. CPRS, Conners parent rating scale; EEG, electroencephalography; hs-CRP, high-sensitivity C-reactive protein; IPAQ, international physical activity questionnaire; MDA, malondialdehyde; NO, nitric oxide

Any possible adverse event will be reported to the Ethical Committee of Shahid Sadoughi University of Medical Sciences and Health Services (Fig. 2). Based on the type and severity of adverse event relate to supplementation, the ethical committee will make a decision on removing the participant or giving an exclusive code or etc. In addition, to evaluate possible toxicity and hypervitaminosis after supplementation with vitamin D, serum level of calcium will be measured at the beginning of the study and after intervention [53].

#### Monitoring data, data accessibility, and confidentially

This RCT will be supervised by a Data Monitoring Committee (DMC). The principal investigator will have access to the final data of the study, and such access for other investigators is limited. There is no plan for granting public access to the full protocol, participant-level dataset, and statistical code. We will present the findings of this study only through the publication. To maintain participant confidentiality, all laboratory samples, reports, data collection, process, and administrative forms will be identified by a coded ID number. All information of participants will be stored securely in locked room with limited access at the study site.

#### Statistical analysis

SPSS version 24 (SPSS, Inc.) will be used for data analysis. The analyses will be performed using the intention-to-treat (ITT) approaches. Using Kolmogorov-Smirnov test, normal distribution of variables will be assessed. Comparing the qualitative variables between two groups will be performed using chi-squared test. To compare the means of normal variables at baseline, at the end of study, and compare the mean changes of normal data between two groups, independent *t*-test will be used. To compare normal variables within the groups, paired sample *t*-test will be applied. Using Mann-Whitney *U* test, comparing abnormal data between the two groups at baseline and after the intervention as well as comparing the mean changes of abnormal data between two groups will be performed. Wilcoxon test will be used to compare the abnormal data within the groups. To control covariates, ANCOVA will be carried out. *P* < 0.05 will be considered significant.

## Discussion

Clinical studies investigating the effect of vitamin D indicate that the overall frequency of side effects related to vitamin D supplementation is low, and most of adverse effects are mild. On the other hand, the prevalence of vitamin D deficiency among patients with ADHD is higher than healthy controls [44]. Moreover, lower levels of vitamin D is associated with higher severity of ADHD symptom [41]. Evidence showed the beneficial effect of vitamin D on central nervous system [29, 54]. RCTs evaluating the effect of vitamin D on behavioral performance of patients with ADHD demonstrated contradictory results [45, 46]. Dehbokri et al. [45] showed vitamin D improves ADHD symptoms with a particular effect on inattention symptoms. However, in the study of Mohammadpour et al. [46], after intervention for 8 weeks, no significant difference between the vitamin D and placebo groups was found in symptoms measured by CPRS. For the first time, the present trial will evaluate the effect vitamin D supplementation on brain waves, NO, MDA, and hs-CRP in patients with ADHD. An important strength of the present study is the assessment of brain waves. However, short duration of the study is an important limitation. The results of the present study will provide a better vision about the effects of vitamin D supplementation in patients with ADHD.

## Trial status

Recruitment of participants began 30 June 2021 and is expected to be completed on 30 August 2021.

## Data Availability

Not applicable.

## Abbreviations

ADHD: Attention deficit/hyperactivity disorder
BMI: Body mass index
CMIA: Chemiluminescent microparticle immunoassay
CNS: Central nervous system
CPRS: Conners’ parent rating scale
DCC: Data coordinating center
DMC: Data Monitoring Committee
DSM-V: Diagnostic and Text Revision of Statistical Manual of Mental Disorders-Fifth Edition
EEG: Electroencephalography
hs-CRP: High-sensitivity C-reactive protein
IL-4: Interleukin 4
IPAQ: International physical activity questionnaire
ITT: Intention-to-treat
MDA: Malondialdehyde
MPH: Methylphenidate
NO: Nitric oxide
ODD: Oppositional defiant disorder
QEEG: Quantitative electroencephalography
SMR: Sensorimotor rhythm
IL-6: Interleukin-6
TNF-α: Tumor necrosis factor-α
VDR: Vitamin D receptor
